# The moderating role of care duration in the relationship between caregiving competence and burden among family caregivers in hospice care

**DOI:** 10.3389/fpubh.2026.1827792

**Published:** 2026-06-16

**Authors:** Ziying Yang, Xiaohui Yu, Cuikui Xu, Dong Zhou, Shujuan Wu, Lijun Wen, Feiyu Lin, Hao Shen, Xiaqiang Wang

**Affiliations:** 1 Medical School of Nantong University, Nantong, China; 2 Department of Oncology, Pingyang Branch, The Second Affiliated Hospital of Wenzhou Medical University, Wenzhou, China; 3 School of Management, Jiangsu University, Zhenjiang, China

**Keywords:** caregiving burden, caregiving competence, family caregivers, hospice care, moderating effect

## Abstract

**Background:**

Caregiving competence is considered an important protective factor in reducing the burden of family caregivers in hospice care. However, whether this relationship is influenced by caregiving duration remains unclear. Previous studies have typically treated caregiving duration as a single quantitative indicator, overlooking potential differences arising from distinct temporal structures.

**Objective:**

To examine the moderating role of caregiving duration in the relationship between caregiving competence and caregiver burden, with particular attention to two temporal structures: daily caregiving hours and continuous caregiving duration.

**Methods:**

Family caregivers of patients receiving hospice care at a secondary hospital in Wenzhou between January 1 and July 31, 2025, were recruited using a convenience sampling method. A total of 345 caregivers participated in the study and completed a general information questionnaire, the Family Caregiver Task Inventory (FCTI), and the Zarit Burden Interview (ZBI-22). Spearman correlation analysis was performed to examine associations among the variables, and the SPSS PROCESS macro was used to test moderating effects.

**Results:**

Caregiving competence was significantly negatively correlated with caregiver burden (r = −0.876, *P* < 0.01). Daily caregiving hours (r = 0.604, *P* < 0.01) and continuous caregiving duration (r = 0.388, *P* < 0.01) were both positively associated with caregiver burden. Moderation analysis showed that daily caregiving hours had no significant moderating effect (B = 0.007, *P* = 0.563), whereas continuous caregiving duration significantly moderated the relationship (B = 0.0270, *P* = 0.004). Simple slope analysis indicated that the protective effect of caregiving competence on burden was stronger among caregivers with shorter caregiving duration (B = −1.609, *P* < 0.001) and weakened as caregiving duration increased (B = −1.374, *P* < 0.001).

**Conclusion:**

The negative association between caregiving competence and burden may be influenced by caregiving time structure. Daily duration did not modify this association, but total cumulative time showed a trend toward a negative association with burden. Thus, cumulative caregiving length, not daily intensity, may be key. Public health practice should focus on long-term family caregivers and explore phased support based on caregiving trajectories to reduce caregiving-related mental health risks and improve hospice care system sustainability.

## 1 Introduction

With the acceleration of global population aging and the continuous evolution of the disease spectrum, the number of patients with end-stage diseases has been steadily increasing. Family caregivers are facing tremendous physical, emotional, and economic burdens, which have become a pressing global public health issue (1, 2). Hospice care represents a comprehensive medical service model for patients with terminal illnesses, aiming to preserve patients' dignity and quality of life at the end of life through symptom management, psychological support, and compassionate care (3). Within this care paradigm, family caregivers assume multifaceted responsibilities encompassing daily assistance, symptom monitoring, and emotional support, rendering them pivotal actors in ensuring care continuity and stability (4). Nevertheless, a substantial body of research indicates that caregivers universally experience varying degrees of physical and psychological burden throughout the caregiving process (5, 6), with their health status potentially deteriorating to levels worse than those of the care recipients themselves (7). This burden not only compromises caregivers' personal wellbeing but may also indirectly affect patients' quality of life by diminishing care quality (8, 9). Therefore, identifying the key factors influencing caregiver burden and exploring effective intervention approaches have become important topics in global hospice care research.

Caregiving competence is commonly conceptualized as a vital resource buffering against caregiver burden. This construct encompasses caregivers' mastery of care-related knowledge, practical competencies, and confidence in managing caregiving situations (10). According to resource buffer theory, personal resources, including knowledge, skills, and self-efficacy, can attenuate the adverse effects of stressors on caregiver burden (11). Extant literature demonstrates that caregivers with greater competence navigate caregiving challenges more effectively, reporting relatively lower burden levels (12, 13). However, a critical unresolved question is whether this protective effect of caregiving competence has a temporal boundary. In other words, does this protective mechanism gradually diminish or even become ineffective as the caregiving trajectory lengthens?

Two competing theoretical perspectives on caregiving time have long coexisted in the literature. Adaptation theory suggests that caregiving burden tends to decrease over time as caregivers adapt to their roles (14). In contrast, fatigue theory posits that prolonged caregiving depletes psychological and physical resources, leading to adaptation fatigue and a decline in coping ability (15). This theoretical divergence implies that caregiving time is not a unidimensional construct but encompasses two distinct temporal structures: daily caregiving intensity and continuous caregiving trajectory (16). The former reflects the immediate “quantity” of caregiving load; the latter represents a cumulative “course effect” over time. Although both are temporal indicators, their underlying mechanisms may differ significantly.

However, existing studies have largely conceptualized caregiving time as a single dimension (17–19), focusing mainly on its association with increased burden, with little attention to the differential effects of distinct temporal structures. This gap has two limitations. Empirically, it precludes distinguishing the moderating effects of daily caregiving intensity from those of continuous caregiving trajectory, limiting accurate understanding of the dynamic between caregiving competence and burden. Clinically, neglect of cumulative caregiving duration obscures the specific risks of long-term caregivers, hindering stratified interventions. In hospice care, distinguishing these two temporal structures is uniquely necessary. Unlike general chronic care, hospice involves end-stage disease and anticipated death. Caregivers face both physical demands and persistent anticipatory grief (20, 21). This dual burden may accelerate psychological resource depletion, causing the protective effect of competence to diminish faster than in other caregiving contexts. Therefore, when caregiving duration is conceptualized through the dual lenses of “intensity” versus “trajectory,” do these dimensions differentially moderate the relationship between caregiving competence and burden? This inquiry elevates caregiving duration from a mere quantitative variable to an analytical construct with inherent structural heterogeneity, potentially illuminating underlying mechanisms in caregiving stress formation.

This question has greater practical relevance in countries and regions with limited hospice care resources. In China, hospice care services remain at an early stage of development, with grassroots institutions facing limited resources, insufficient staffing, and a lack of systematic support for family caregivers. In this context, caregivers often undertake heavy tasks over prolonged periods. Nevertheless, whether their psychological resources continuously deplete as caregiving time extends, and how this depletion affects the buffering effect of caregiving competence on burden, require empirical investigation. Accordingly, this study focuses on family caregivers in the Chinese hospice care context, using a questionnaire survey to examine the moderating roles of daily caregiving duration and continuous caregiving time in the competence–burden relationship, with the goal of informing support strategies for family caregivers in resource-limited hospice care settings worldwide.

## 2 Methods

### 2.1 Study design

A convenience sampling method was adopted to recruit family caregivers of patients receiving hospice care at a secondary hospital in Wenzhou City between January 1 and July 31, 2025, with 345 participants ultimately included. This hospital serves as the primary provider of county-level medical services, and the hospice care patients it admits exhibit strong representativeness in terms of disease characteristics, socioeconomic status, and caregiving models when compared to those in other domestic medical institutions. Accordingly, the findings may offer meaningful insights for healthcare settings characterized by limited hospice care resources.

#### 2.1.1 Inclusion criteria

Patients: Aged ≥18 years; meeting the diagnostic criteria for terminal illness, as referenced in the hospice Care Practice Guidelines (Trial) (22) and expert consensus (23), including but not limited to advanced cancer, decompensated stage of chronic organ failure, and advanced neurodegenerative diseases. All patient-related data in this study were derived from the hospital's clinical medical information system. Caregivers: Aged ≥18 years; having a blood relationship, marital relationship, or legally recognized kinship with the patient; assuming primary caregiving responsibilities during the patient's hospitalization (if multiple caregivers are involved, the one with the longest daily caregiving duration is selected); providing informed consent and voluntarily participating in this study.

#### 2.1.2 Exclusion criteria

Paid caregivers with an employment relationship; caregivers with mental disorders or cognitive communication impairments (assessed by inquiring about prior medical diagnoses and evaluating their ability to understand and cooperate through on-site communication); caregivers currently participating in other clinical studies; caregivers who have recently experienced major negative events (e.g., bankruptcy, bereavement, or various natural disasters).

#### 2.1.3 Sample size calculation

Sample size estimation was conducted using G^*^Power 3.1 software (24). The “Linear multiple regression: Fixed model, R^2^ increase” model under the F test was selected, with parameters set as follows: effect size f^2^ = 0.05, α = 0.05, and statistical power (1-β) = 0.90. The regression model included 19 predictor variables (16 control variables, one independent variable, and two moderating variables), with four interaction terms to be tested. The calculated minimum required sample size was 294. After accounting for a 10% invalid response rate, the target sample size was set at a minimum of 324.

A total of 360 questionnaires were distributed, and 345 valid questionnaires were returned, representing an effective response rate of 95.8%. A *post hoc* power analysis based on the observed interaction effect (β = 0.027, corresponding to f^2^ = 0.03) produced a statistical power of 0.86, suggesting that the achieved sample size had sufficient statistical power.

### 2.2 Research instruments

① General Information Questionnaire: The questionnaire was self-designed based on clinical nursing practice and consultation with experts in relevant fields. It included patient characteristics (sex, age, disease type, and physical self-care status) as well as caregiver characteristics (sex, age, educational level, relationship with the patient, daily caregiving duration, and total caregiving duration). All data collection procedures complied with research ethical requirements, and no sensitive personally identifiable information (e.g., names, identification numbers) was collected.

② Performance Status Assessment Scale: This study used the Eastern Cooperative Oncology Group Performance Status (ECOG PS) to assess patients‘ functional status (25). The ECOG PS is a routine inpatient assessment, and data were obtained from the clinical medical information system. The score is assigned by the attending physician based on the patient's activities of daily living and self-care ability. Scores range from 0 to 5: 0 = normal activity; 1 = symptomatic but almost completely ambulatory; 2 = sometimes bedridden but ambulatory >50% of the day; 3 = frequently bedridden, in bed >50% of the day; 4 = completely bedridden; 5 = dead. Higher scores indicate poorer performance status. In this study, ECOG PS was used to describe patients' functional status and served as a background variable in subsequent analyses of caregiver competence and burden (27).

③ Family Caregiver Task Inventory (FCTI) (26): The scale was originally developed by Clark and Rakowski (52). The present study adopted the Chinese version adapted by Lee et al. (54) from Hong Kong. The Cronbach's α coefficient of this version was 0.930, indicating good reliability and validity. The scale was used for non-commercial academic research, and we confirm that its use complied with the relevant requirements and regulations. The scale comprises five dimensions: adapting to caregiving roles, coping with needs and providing assistance, managing personal emotions, evaluating family and community resources, and adjusting life to meet caregiving needs. Each dimension contains 5 items, totaling 25 items. The scale uses a Likert 3-point scoring system, ranging from 0 to 2 points (0 = “not difficult”; 1 = “difficult”; 2 = “very difficult”). The total score ranges from 0 to 50, with higher scores indicating poorer comprehensive caregiving competence and greater difficulty in undertaking caregiving tasks. To facilitate result interpretation, the total score of the FCTI was reverse-coded prior to analysis (i.e., new score = 50—original score). After reverse coding, higher scores indicated greater caregiving competence. All correlation and regression analyses were performed based on the reverse-coded data. In this study, the Cronbach's α coefficient of this scale was 0.837.

④ Zarit Burden Interview (ZBI): Originally developed by Zarit et al. (53), the scale was used in its Chinese version adapted by Wang Lie and colleagues (55). The Cronbach's α was 0.870, demonstrating satisfactory reliability and validity. The scale was employed for non-commercial academic research, and its use was confirmed to comply with applicable requirements and guidelines. The scale consists of 22 items covering three dimensions: responsibility, personal burden, and role burden. The scale uses a Likert 5-point scoring system (0 = “never”; 1 = “rarely”; 2 = “sometimes”; 3 = “frequently”; 4 = “nearly always”), with total scores ranging from 0 to 88. Higher scores indicate greater caregiver burden. In this study, the Cronbach's α coefficient of this scale was 0.929.

### 2.3 Data collection

Data collection was performed by trained nurses from the hospice care unit of the secondary hospital. Family caregivers of eligible hospitalized hospice care patients were recruited to complete a face-to-face questionnaire, with each interview lasting approximately 20–30 min. Prior to participation, caregivers were informed of the study's purpose and procedures, and written informed consent was obtained. Questionnaires were self-administered. The questionnaires were completed independently by the participants. For those unable to complete them independently due to visual impairment, educational level, or other reasons, the investigators read the questions aloud one by one in a neutral manner, avoiding suggestive guidance, and recorded the answers based on the participants' responses. Completed questionnaires were collected on-site, and the investigators carefully checked each questionnaire for completeness. Any missing items were promptly supplemented by the participants.

**Figure 1:**
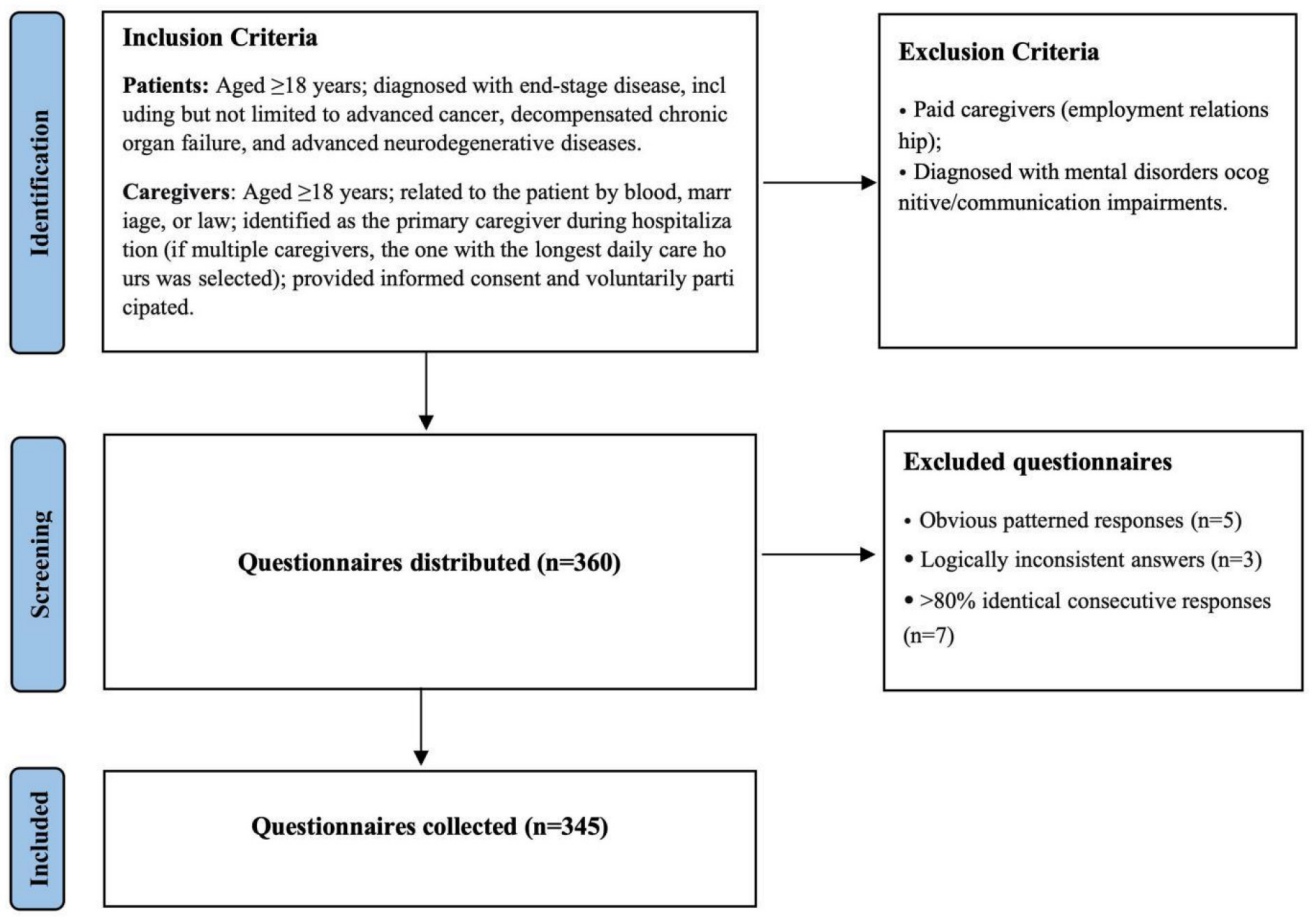
Flowchart of questionnaire screening.

Participants were enrolled consecutively until both the sample size estimation requirements and the statistical analysis needs were met. A total of 360 questionnaires were distributed, and 345 valid questionnaires were recovered, yielding an effective response rate of 95.8%. The sample screening process is illustrated in Figure 1.

### 2.4 Statistical methods

Data collation and statistical analyses were performed using SPSS version 27.0 (IBM Corp., Armonk, NY, USA). For measurement data, normally distributed variables were expressed as mean ± standard deviation (X±S), while skewed distributions were presented as median and interquartile range. Count data were described using frequencies and constituent ratios [N (%)]. Spearman's rank correlation analysis in SPSS was employed to examine the correlations among caregiving time, caregiving competence, and caregiver burden. Prior to conducting moderation analysis, the independent variable (caregiving competence) and the moderating variables (daily caregiving duration and continuous caregiving time) were mean-centered to reduce non-essential multicollinearity between the interaction terms and the main effects.

Moderation analyses were conducted using the PROCESS macro (Version 4.0) developed by Rockwood and Hayes (51). Daily caregiving duration and continuous caregiving time were examined as moderators. Model 1 (basic moderation model) and Model 3 (moderation model with two moderators) were selected to test the moderating effects on the relationship between caregiving competence and burden. Bias-corrected bootstrapping with 5,000 resamples was used to estimate standard errors and 95% confidence intervals. For interaction terms that reached statistical significance, simple slope analyses were further performed to elucidate the specific patterns of the moderating effects. A two-tailed *P*-value < 0.05 was considered statistically significant for all analyses.

### 2.5 Ethical review and quality control

This study complied with the principles of the Declaration of Helsinki (28) and was approved by the Ethics Committee of the Second People's Hospital of Pingyang County (Approval No. 2024036). All participants provided written informed consent. A pilot study involving 20 caregivers was conducted prior to the formal survey to optimize the wording of the questionnaire. During the data collection phase, trained research assistants used standardized instructions to administer the questionnaires, which were completed on site, collected immediately, and reviewed item by item to ensure data completeness. Data management was performed with double independent data entry, with one of the two entry persons having a background in data analysis. After entry, consistency checks were conducted, and any discrepancies were traced back to the original data for correction. The final database was locked for statistical analysis.

## 3 Results

### 3.1 Demographic characteristics of the study participants

As shown in Table 1, among the patients, 261 (75.7%) were male and 84 (24.3%) were female, with a mean age of 70.70 ± 10.47 years. The predominant disease types were digestive system diseases (41.7%) and respiratory system diseases (29.3%), and 61.7% of patients had a confirmed disease course of >12 months. Married patients accounted for 85.2%, and 83.5% were covered by the New Rural Cooperative Medical Scheme. The ECOG performance status scores were predominantly 2 (55.4%) and 3 (36.8%).

**Table 1:** Sociodemographic characteristics of study participants.

Category	Variable	*N* (%)/X ±S
Patient	Gender	
	Male	261 (75.7%)
	Female	84 (24.3%)
	Age	70.70 ± 10.47
	Marriage	
	Unmarried	6 (1.7%)
	Married	294 (85.2%)
	Divorced	6 (1.7%)
	Widowed	39 (11.3%)
	Medical payment	
	Employee medical insurance	15 (4.3%)
	Urban and rural residents' medical insurance	41 (11.9%)
	The new rural cooperative medical insurance	289 (83.8%)
	Disease type	
	Respiratory system	101 (29.3%)
	Digestive system	144 (41.7%)
	Hematologic system	30 (8.7%)
	Urinary system	14 (4.1%)
	Reproductive system	39 (11.3%)
	Head and neck	11 (3.2%)
	Other	6 (1.7%)
	Time since diagnosis (months)	
	< 6	87 (25.2%)
	6 12	45 (13.0%)
	>12	213 (61.7%)
	ECOG	
	1	16 (4.6%)
	2	191 (55.4%)
	3	127 (36.8%)
	4	11 (3.2%)
Caregiver	Gender	
	Male	98 (28.4%)
	Female	247 (71.6%)
	Age	58.44 ± 12.36
	Marriage	
	Unmarried	11 (3.2%)
	Married	329 (95.4%)
	Divorced	5 (1.4%)
	Education	
	Primary school or below	167 (48.4%)
	Junior high school	141 (40.9%)
	High school/technical secondary school	26 (7.5%)
	College degree or above	11 (3.2%)
	Employment status	
	Employed	169 (49.0%)
	Retired/unemployed	176 (51.0%)
	Monthly income (RMB)	
	< 1,000	44 (12.8%)
	1,000–3,000	77 (22.3%)
	3,000–5,000	116 (33.6%)
	>5,000	108 (31.3%)
	Relationship to patient	
	Spouse	190 (55.1%)
	Child	144 (41.7%)
	Sibling	5 (1.4%)
	Other (e.g., nephews, nieces, grandchildren, etc.)	6 (1.7%)
	Co-caregiver	
	Yes	305 (88.4%)
	No	40 (11.6%)
	Comorbid illness	
	Yes	163 (47.2%)
	No	182 (52.8%)
	Daily caregiving hours (hours)	17.54 ± 3.21
	Duration of continuous care (months)	10.17 ± 4.60

ECOG, Eastern Cooperative Oncology Group performance status score; RMB, Renminbi.

Among the caregivers, 247 (71.6%) were female and 98 (28.4%) were male, with a mean age of 58.44 ± 12.36 years. The majority were married (95.4%), and 51.0% were retired or unemployed. Regarding educational level, caregivers with primary school education or below accounted for 48.4%, and those with junior middle school education accounted for 40.9%. The primary relationships with patients were spouse (55.1%) and child (41.7%). The mean daily caregiving duration was 17.54 ± 3.21 h, and the mean cumulative caregiving duration was 10.17 ± 4.60 months. Additionally, 88.4% of caregivers had other individuals sharing caregiving responsibilities, and 47.2% had comorbid chronic diseases.

### 3.2 Distribution of family caregivers' caregiving competence and burden scores

In this study, the total score for family caregivers' caregiving competence was 29.0 (24.0, 31.0). The total score for caregiver burden was 49.0 (45.0, 60.0). Scores for each dimension of caregiving competence ranged from 3.0 to 9.0 points. Among these dimensions, the dimension of managing personal emotions had the lowest score, while the dimension of evaluating family and community resources had the highest score. Regarding the dimensions of caregiver burden, the personal burden dimension had the highest score, followed by the responsibility burden dimension, and the role burden dimension had the lowest score. Detailed scores for each dimension are presented in Table 2.

**Table 2:** Scores of caregiving competence and burden among family caregivers.

Scale	Dimension	Items	Min	Max	Total [median (IQR)]
FCTI	Caregiving competence	25	19	39	29.0 (24.0, 31.0)
	Adapting to the caregiving role	5	3	8	6.0 (5.0, 7.0)
	Responding to needs and providing assistance	5	3	8	5.0 (5.0, 6.0)
	Managing personal emotions	5	3	8	5.0 (4.0, 6.0)
	Evaluating family and community Resources	5	3	9	7.0 (6.0, 7.0)
	Adjusting life to meet caregiving Needs	5	3	9	6.0 (5.0, 6.0)
ZBI	Caregiver burden	22	34	64	49.0 (45.0, 60.0)
	Personal burden	12	16	39	26.0 (22.0, 33.5)
	Responsibility burden	6	8	15	12.0 (10.0, 13.0)
	Role burden	4	9	13	11.0 (10.0, 12.0)

FCTI, Family Caregiver Task Inventory; ZBI, Zarit Burden Interview.

### 3.3 Correlation analysis of caregiver burden, competence, and caregiving time

Spearman correlation analysis (Table 3) revealed that caregiving competence was significantly negatively correlated with caregiver burden (r = −0.876, *P* < 0.01), indicating that caregivers with lower competence perceived greater burden. Both daily caregiving duration and continuous caregiving duration were significantly positively correlated with caregiver burden (r = 0.604 and 0.388, respectively, *P* < 0.01), suggesting that greater time investment in caregiving was associated with higher burden.

**Table 3:** Correlation analysis of caregiving competence, time input, and caregiving burden.

Variable	ZBI	FCTI	Daily caregiving duration	Continuous caregiving duration
ZBI	1.000	-	-	-
FCTI	−0.876^**^	1.000	-	-
Daily caregiving duration	0.604^**^	−0.564^**^	1.000	-
Continuous caregiving duration	0.388^**^	−0.327^**^	0.454^**^	1.000

^**^P < 0.01;—indicates data duplication, not listed again.

In this study, although the correlation coefficient between caregiving competence and burden was relatively high, the two temporal dimensions were moderately positively correlated (r = 0.454, *P* < 0.01), indicating that they are related but distinguishable. The variance inflation factor (VIF) for each variable was less than 5, suggesting no serious multicollinearity issue and that the variables could be further included in regression analyses.

### 3.4 Moderation effect analysis of caregiving time on the competence-burden relationship

#### 3.4.1 Moderation effect of daily caregiving duration

Model 1 in the PROCESS macro was employed, with caregiver burden as the dependent variable (assigned value: measured value), caregiving competence as the independent variable, and daily caregiving duration as the moderating variable. Demographic variables, including patients' functional status, age, and gender, were included in the regression equation as control variables (assigned values: dummy variables for categorical variables, measured values for continuous variables).

The results (Table 4) showed that the model explained a substantial proportion of the variance (R^2^ = 0.86, *p* < 0.001). Caregiving competence significantly and negatively predicted caregiving burden (B = −1.480, *p* < 0.001), whereas daily caregiving duration significantly and positively predicted caregiving burden (B = 0.340, *p* < 0.001). The interaction term between caregiving competence and daily caregiving duration did not reach statistical significance (B = 0.007, *p* = 0.563), indicating that daily caregiving duration did not have a moderating effect on the relationship between caregiving competence and burden.

**Table 4:** Moderating effect of daily care hours on the relationship between caregiving competence and burden.

Variable	*B*	*Boot SE*	*t*	*p*	95% *Boot CI*
Constant	53.800^**^	3.680	13.830	< 0.001	46.490 60.760
FCTI	−1.480^**^	0.050	−27.850	< 0.001	−1.580 −1.390
Daily caregiving duration	0.340^**^	0.080	4.160	< 0.001	0.190 0.500
Caregiving competence ^*^daily caregiving duration	0.007	0.010	0.580	0.563	−0.012 0.027

R^2^ = 0.860, F_(19, 325)_ = 105.020^**^. ^**^P < 0.01; Boot SE and Boot CI were estimated based on 5,000 bootstrap resamples; PROCESS Model 1 was used for moderation analysis. DV, caregiver burden (ZBI); IV, caregiving competence (FCTI); moderator, daily caregiving duration; interaction, FCTI × daily caregiving duration. Covariates (*n* = 16): patient characteristics (sex, age, marital status, insurance type, disease type, time since diagnosis, ECOG PS) and caregiver characteristics (sex, age, marital status, education, employment, monthly income, relationship to patient, co-caregivers, caregiving while ill). Full model included 19 predictors (16 covariates + IV + moderator + interaction).

#### 3.4.2 Moderation effect of continuous caregiving duration

Using continuous caregiving duration as the moderating variable, the same method was employed to examine its moderating effect on the relationship between caregiving competence and burden. The results (Table 5) showed that the model accounted for a high proportion of variance (R^2^ = 0.860, *p* < 0.001). Caregiving competence significantly and negatively predicted caregiver burden (B = −1.550, *p* < 0.001), while continuous caregiving duration significantly and positively predicted caregiver burden (B = 0.230, *p* < 0.001). The interaction term between caregiving competence and continuous caregiving duration was statistically significant (B = 0.027, *p* = 0.004), indicating that continuous caregiving duration significantly moderated the relationship between caregiving competence and burden.

**Table 5:** Moderating effect of continuous care duration on the relationship between caregiving competence and burden.

Variable	*B*	*Boot SE*	*t*	*p*	95% *Boot CI*
Constant	52.400^**^	3.510	13.540	< 0.001	45.490 59.230
FCTI	−1.550^**^	0.040	−31.290	< 0.001	−1.630 −1.470
Continuous caregiving duration	0.230^**^	0.050	4.750	< 0.001	0.130 0.320
Caregiving competence ^*^continuous caregiving duration	0.027^*^	0.0076	2.880	0.004	0.012 0.042

R^2^ = 0.860, F_(19, 325)_ = 108.210^**^. ^**^P < 0.01; Boot SE and Boot CI were estimated based on 5,000 bootstrap resamples; PROCESS Model 1 was used for moderation analysis. DV, caregiver burden (ZBI); IV, caregiving competence (FCTI); moderator, continuous caregiving time; interaction, FCTI × continuous caregiving time. Covariates (*n* = 16): patient characteristics (sex, age, marital status, insurance type, disease type, time since diagnosis, ECOG PS) and caregiver characteristics (sex, age, marital status, education, employment, monthly income, relationship to patient, co-caregivers, caregiving while ill). Full model: 19 predictors (16 covariates + IV + moderator + interaction).

**Figure 2:**
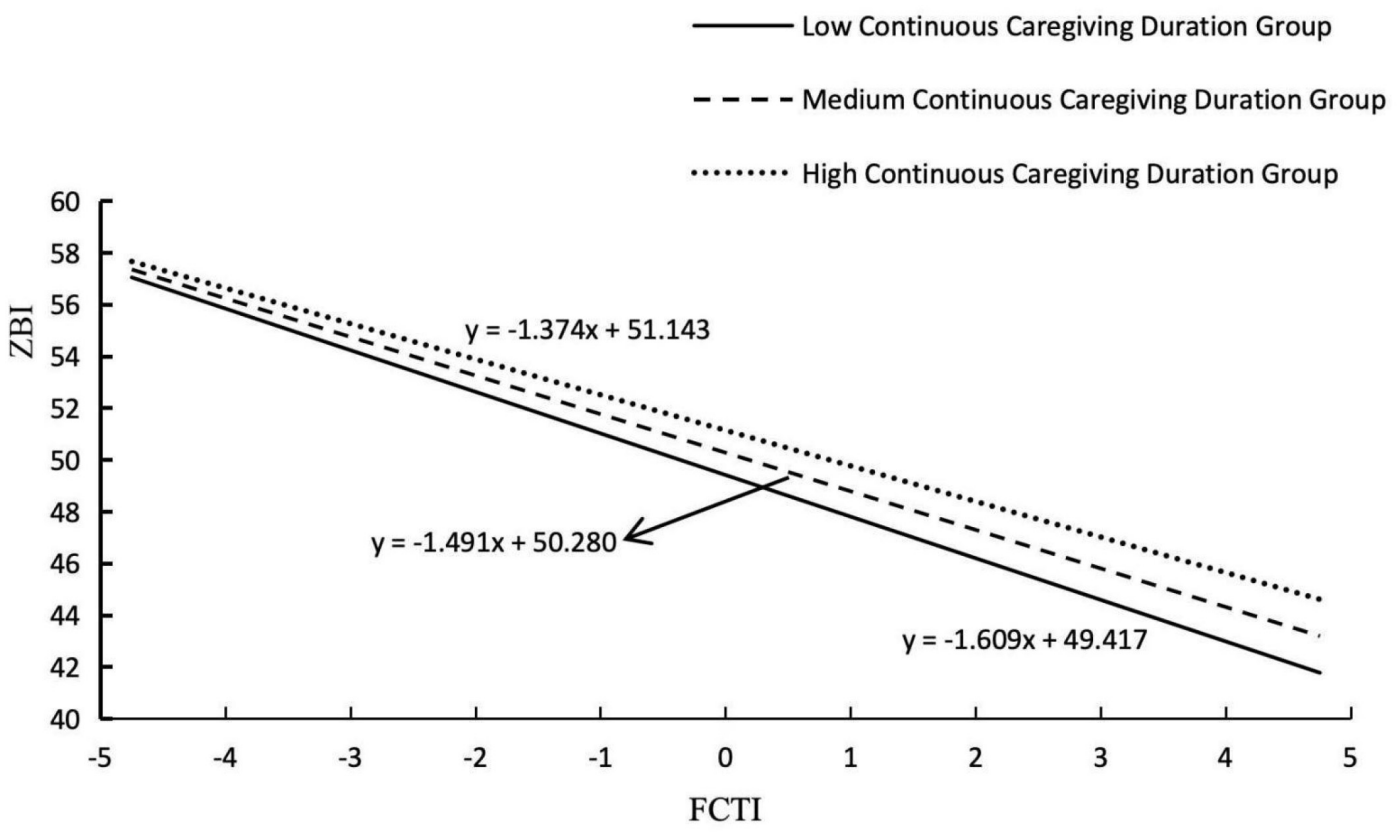
Simple slope plot of the moderating effect of continuous care duration.

Simple slope analysis revealed (Figure 2) that when continuous caregiving duration was short, the negative effect of caregiving competence on burden was stronger (B = −1.609, *p* < 0.001); when continuous caregiving duration was long, this effect was attenuated (B = −1.374, p < 0.001). This indicates that continuous caregiving duration significantly moderates the relationship between caregiving competence and burden: the longer the caregiving trajectory, the more the protective effect of caregiving competence is weakened. Table 6 shows the predicted burden values by level of continuous caregiving duration.

**Table 6:** Predicted values of caregiving burden by caregiving competence at different levels of continuous care duration.

FCTI	Low continuous caregiving duration group	Medium continuous caregiving duration group	High continuous caregiving duration group
Low	57.20	57.65	58.10
Medium	49.25	50.29	51.33
High	41.30	42.93	44.56

Low, medium, and high caregiving competence correspond to mean ± 1 standard deviation, respectively; low, medium, and high continuous caregiving duration groups correspond to mean ± 1 standard deviation, respectively.

To further verify the reliability of the moderating effect of continuous caregiving duration, daily caregiving duration was included as a control variable, and the significance of the interaction between caregiving competence and continuous caregiving duration was re-examined. As shown in Table 7, the model explained a substantial proportion of the variance (R^2^ = 0.870, *p* < 0.001), and the interaction term remained significant (B = 0.026, *p* = 0.006), indicating that the moderating effect of continuous caregiving time was independent of daily caregiving duration and demonstrated good robustness.

**Table 7:** Moderating effect of continuous care duration on the relationship between caregiving competence and burden.

Variable	*B*	*Boot SE*	*t*	*p*	95% *BootCI*
Constant	47.920^**^	3.740	11.760	<0.001	40.690 55.250
FCTI	−1.490^**^	0.040	−28.490	<0.001	−1.580 −1.400
Continuous caregiving duration	0.190^**^	0.050	3.870	<0.001	0.090 0.280
Daily caregiving duration	0.260^**^	0.080	3.140	0.002	0.110 0.410
Caregiving competence^*^ continuous caregiving duration	0.026^**^	0.008	2.760	0.006	0.011 0.041

R^2^ = 0.870, F_(20, 324)_ = 106.100^**^. ^**^P <0.01; Boot SE and Boot CI were estimated based on 5,000 bootstrap resamples; PROCESS Model 3 was used for moderation analysis. DV, caregiver burden (ZBI); IV, caregiving competence (FCTI); moderators, continuous caregiving time and daily caregiving duration; interaction, FCTI × continuous caregiving time. Covariates (*n* = 16): patient characteristics (sex, age, marital status, insurance type, disease type, time since diagnosis, ECOG PS) and caregiver characteristics (sex, age, marital status, education, employment, monthly income, relationship to patient, co-caregivers, caregiving while ill). Full model: 20 predictors (16 covariates + IV + 2 moderators + interaction).

#### 3.4.3 Synergistic moderating effect of daily caregiving duration and continuous caregiving duration

Model 3 in the PROCESS macro was used to examine the joint moderating effects of daily caregiving duration and continuous caregiving time. The results are presented in Table 8. The model explained a substantial proportion of the variance (R^2^ = 0.87, *p* < 0.001). The interaction term between caregiving competence and continuous caregiving time was not significant (B = 0.024, *p* = 0.100), nor were the interactions between caregiving competence and daily caregiving duration (B = −0.004, *p* = 0.777) or between continuous caregiving time and daily caregiving duration (B = −0.013, *p* = 0.403). The three-way interaction among the three variables also did not reach statistical significance (B = −0.003, *p* = 0.338). These results indicate that there was no synergistic moderating effect between daily caregiving duration and continuous caregiving time; that is, the moderating effect of continuous caregiving time did not vary as a function of daily caregiving duration.

**Table 8:** Synergistic moderating effect of daily care hours and continuous care duration.

Variable	*B*	*Boot SE*	*t*	*p*	95% *Boot CI*
Constant	51.700^**^	3.650	13.360	< 0.001	44.390 58.730
FCTI	−1.480^**^	0.040	−26.980	< 0.001	−1.570 −1.400
Continuous caregiving duration	0.170^**^	0.06	3.030	0.003	0.050 0.280
Daily caregiving duration	0.230^**^	0.090	2.600	0.010	0.060 0.400
Caregiving competence^*^ continuous caregiving duration	0.024	0.013	1.650	0.100	−0.001 0.051
Caregiving competence^*^ daily caregiving duration	−0.004	0.012	−0.280	0.777	−0.026 0.019
Continuous caregiving duration^*^ daily caregiving duration	−0.013	0.016	−0.840	0.403	−0.045 0.017
Caregiving competence^*^ continuous caregiving duration^*^ daily caregiving duration	−0.003	0.003	−0.960	0.338	−0.008 0.002

R^2^ = 0.870, F_(23, 321)_ = 91.95^**^. ^**^P < 0.01; Boot SE and Boot CI were estimated based on 5,000 bootstrap resamples; PROCESS Model 3 was used for moderation analysis. DV, caregiver burden (ZBI); IV, caregiving competence (FCTI); moderators, continuous caregiving time (CCT) and daily caregiving duration (DCD). Interaction terms: FCTI × CCT, FCTI × DCD, CCT × DCD, and FCTI × CCT × DCD. Covariates (*n* = 16): patient characteristics (sex, age, marital status, insurance type, disease type, time since diagnosis, ECOG PS) and caregiver characteristics (sex, age, marital status, education, employment, monthly income, relationship to patient, co-caregivers, caregiving while ill). Full model: 24 predictors (16 covariates + IV + 2 moderators + 4 interaction terms).

## 4 Discussion

### 4.1 Current status of caregiving competence and burden among family caregivers in hospice care

Our findings indicate that family caregivers in hospice care possess moderate overall caregiving competence, with significant disparities across different dimensions. Notably, emotional management capacity emerged as the weakest domain, a finding largely consistent with Lowers et al. (29). In the context of hospice care, caregivers are not only required to undertake complex daily caregiving tasks but also to confront the prolonged process of progressive patient deterioration and the dying process. The resulting anticipatory grief, anxiety, and emotional exhaustion may be closely related to difficulties in emotion regulation, suggesting that emotion regulation may be a relatively weak component of caregiving competence (30). From a public health perspective, deficits in emotion regulation may not only affect caregivers‘ own mental health but may also influence patients' emotional experiences and the quality of end-of-life care through caregiving interactions. Thus, emotion regulation difficulties can be considered a potential factor compromising the stability of family caregiving systems.

Regarding caregiving burden, this study found that the personal burden subscale scored the highest, suggesting that the caregiving role may significantly compress caregivers' personal living space and social roles. This finding is consistent with previous research on “role captivity” (31, 32), which indicates that when caregiving responsibilities gradually occupy an individual's primary life role, caregivers may experience a marked reduction in personal time, autonomy, and opportunities for social participation, potentially exerting negative effects on their subjective wellbeing and mental health. From a public health perspective, such role pressure may not only manifest as a decline in quality of life at the individual level but may also place caregivers in a state of chronic stress, increasing the risk of mental health problems such as depression, anxiety, and emotional exhaustion, thereby potentially affecting the sustainability of family caregiving systems.

Therefore, our results highlight the necessity for systematic interventions addressing emotional regulation and role strain, beyond traditional caregiving skills support, within the hospice care setting. Future intervention strategies should integrate psychological support services, caregiver emotional management training, and the development of social support networks. This multifaceted approach aims to enhance caregiving competence while mitigating the long-term psychological and social stressors experienced by caregivers.

### 4.2 Moderating mechanisms of caregiving duration in the relationship between caregiving competence and burden

Building on existing research, this study further examined the moderating role of caregiving duration in the relationship between caregiving competence and caregiver burden. Results revealed a significant negative correlation between caregiving competence and burden: higher ability levels were associated with lower perceived burden. This finding is consistent with previous reports (12, 33). However, further analysis demonstrated that different temporal structures of caregiving duration exerted distinct moderating effects on this relationship.

Daily caregiving hours did not significantly moderate the relationship between caregiving competence and burden. Daily caregiving hours primarily reflect care intensity, representing the level of care investment per unit time. Our findings indicate that the buffering effect of caregiving competence on burden remained relatively stable across different intensity conditions: caregivers with higher ability consistently reported lower burden levels than those with lower ability, regardless of daily time investment. This suggests that caregiving competence possesses a certain “cross-intensity stability,” meaning that individual resources can sustain their buffering function across varying levels of caregiving stress.

Continuous caregiving duration, which refers to the length of time individuals had assumed the caregiving role, demonstrated a significant moderating effect. In this study, as caregiving time extended, the negative association between caregiving competence and caregiving burden showed a gradually weakening trend. This finding suggests that the caregiving trajectory itself may have an important cumulative temporal effect, and that long-term caregiving situations may be accompanied by a gradual depletion of caregivers' psychological and social resources.

This finding aligns with Schulz et al.'s (15) concept of “adaptation fatigue” among long-term caregivers. Sustained stress in prolonged caregiving contexts may gradually exceed individuals' resource recovery capacity, rendering even caregivers with high ability unable to fully resist the negative impacts of chronic stress. Kim et al. (34) similarly found that as patients' disease progressed, caregivers' psychological resources showed continuous depletion trends, with emotional exhaustion levels rising steadily. Gallagher et al. (35) further noted that long-term caregiving may alter caregivers' perceptions of their caregiving role, sharpening their awareness of disease irreversibility and future uncertainty, thereby increasing emotional distress.

Notably, after controlling for daily caregiving hours, the moderating effect of continuous caregiving duration remained significant, and no synergistic effect emerged between the two temporal variables. This indicates that caregiving duration is not a unitary quantitative indicator but encompasses distinct temporal structures: daily caregiving hours primarily reflect immediate stress, whereas continuous caregiving duration represents accumulated long-term stress. These two dimensions play different roles in the formation of caregiving stress.

### 4.3 Temporal boundaries of the protective effect of caregiving competence and their theoretical implications

This study further proposes the concept of a “temporal boundary of the protective effect of caregiving competence,” which refers to the tendency for the buffering effect of caregiving competence on burden to attenuate as continuous caregiving time extends. To identify this temporal boundary, we constructed an interaction term between caregiving competence and continuous caregiving time, tested its significance to determine whether a temporal boundary existed, and used simple slope analysis to examine whether the effect of caregiving competence declined over time. The results showed that the interaction term was significant and that the effect size of caregiving competence decreased as continuous caregiving time increased, confirming the existence of a temporal boundary.

Existing research has typically conceptualized caregiving competence as a stable individual resource, assuming that it exerts a sustained protective effect throughout the caregiving process (36, 37). However, the present study found that the association between caregiving competence and burden exhibited a phase-specific pattern: in the early stage of the caregiving trajectory, caregiving competence was associated with lower levels of burden; as caregiving time extended, this negative association gradually weakened. This phenomenon can be explained by the conservation of resources theory, which posits that when individual resources are depleted over time without adequate replenishment, individuals enter a state of resource exhaustion, accompanied by a decline in coping ability (38). Integrating this theory with the caregiver stress process model reveals that while the stress process model emphasizes the buffering role of coping resources, the conservation of resources theory elucidates the cumulative mechanism of resource depletion. On this basis, the present findings introduce a temporal dimension to the stress process model.

Previous research has supported the above findings. Atefi et al. (39) found that caregivers' sense of competence significantly declined over time. Lou et al. (40) identified distinct caregiver stress trajectories, with the subgroup experiencing persistently increasing stress also exhibiting declines in caregiving competence and coping resources. Kayaalp et al. (41) demonstrated that caregiving stress continuously affects caregivers' mental health through work–family conflict. Collectively, these findings provide a complementary perspective to the classic caregiver stress process model (42)—namely, that the role of caregiving resources may change over the course of the caregiving trajectory. By proposing the concept of a “temporal boundary,” this study incorporates the temporal dimension into the analytical framework of caregiver stress mechanisms, offering a theoretical basis for understanding the dynamic nature of caregiving competence.

### 4.4 Implications and recommendations for hospice clinical practice

Firstly, incorporate caregiving trajectory into routine risk screening frameworks. Current clinical assessments predominantly focus on the immediate impact of caregiving intensity, with insufficient attention paid to the cumulative effects of caregiving duration. It is recommended that continuous caregiving time be used as a basic reference indicator for risk stratification of caregivers, with priority monitoring and early intervention implemented for those who have undertaken long-term caregiving roles (1) In terms of risk classification, the four risk profiles of the CAREPAL-8 screening tool (43) from Germany, including currently stable, unmet needs, psychological burden, and high risk, may serve as a useful reference. A dynamic risk stratification system based on the caregiving trajectory could be established, which would enable the matching of differentiated intervention strategies according to caregivers' risk levels, thereby facilitating the transition from universal screening to tiered and categorized management. (2) Regarding assessment content, the multidimensional assessment model of the Australian Caregiver Health Support Program (44) may serve as a useful reference. By comprehensively evaluating indicators such as burden level, caregiving competence, physical and mental health, and social support, the multidimensional needs of caregivers can be fully identified, thereby avoiding the limitations of single-indicator assessment. (3) In terms of management mechanisms, the approach of the stratified bereavement support intervention checklist (45) may serve as a useful reference. A closed-loop management process comprising assessment, stratification, follow-up, and adjustment could be established, which would provide practical guidance for transitioning from single-screening to longitudinal monitoring.

Secondly, develop a family caregiver empowerment training system. Caregiving competence training should be integrated into routine services as a foundational intervention. Training content should prioritize emotional management while encompassing symptom management, communication skills, and resource acquisition, thereby enhancing caregivers' sense of caregiving competence. Training models can draw from multidisciplinary collaboration approaches (46), adopting a three-tier team structure of “core-executive-auxiliary” to deliver stratified training for caregivers. Training modalities should be diversified, combining online and offline formats to improve accessibility and flexibility (47, 48). Throughout this process, empowerment education principles (49) should be incorporated to stimulate caregivers' intrinsic potential and self-efficacy, transforming them from passive executors into active problem-solvers. Additionally, drawing on the experience of the “PalliActive Caregivers” intervention program (50), a robust training effectiveness evaluation mechanism should be established to continuously optimize training protocols.

Thirdly, establish a support framework based on caregiving trajectory and ability level. Caregivers can be categorized into four types based on caregiving duration and ability level, with corresponding support strategies: (1) Novice caregivers with low ability: focus on skill training to build caregiving confidence and mitigate burden at its source; (2) Novice caregivers with high ability: provide preventive support to guard against long-term resource depletion; (3) Long-term caregivers with low ability: constitute a high-risk population requiring comprehensive, intensive support encompassing skill training, psychological counseling, and social resource linkage; (4) Long-term caregivers with high ability: shift intervention focus toward emotional exhaustion identification and psychological support, helping them rebuild personal boundaries and restore individual life space. Concurrently, a periodic assessment mechanism should be established to dynamically adjust support levels based on changes in caregivers' status, transitioning from “one-size-fits-all” services to “precision-based” stratified support.

### 4.5 Limitations and future research

Although rigorous quality control measures were implemented in study design, sample selection, and data analysis, several limitations should be acknowledged.

Firstly, the cross-sectional design precludes causal inferences regarding the relationships among caregiving time, caregiving competence, and caregiving burden. In particular, in moderation analysis, interaction terms can only reveal associative patterns among variables and cannot establish temporal order or causal direction. Moreover, the high negative correlation between the FCTI and ZBI (r = −0.876) suggests potential conceptual overlap between the two measures, indicating that caregiving competence and caregiving burden may not be fully distinguishable in empirical measurement. Future studies could employ longitudinal designs and structural equation modeling to further examine causal directions and discriminant validity of the scales.

Secondly, the convenience sampling method may have introduced selection bias, limiting the generalizability of the findings. Future multicenter studies with larger sample sizes and random sampling methods are warranted to enhance sample representativeness and the generalizability of conclusions. In addition, caregiving time was self-reported, which may be subject to recall bias. Future research could incorporate objective recording methods such as caregiving logs or use qualitative interviews to gain a deeper understanding of caregivers' subjective experiences of caregiving time.

Finally, this study did not explore the mediating mechanisms through which continuous caregiving time depletes the protective effect of caregiving competence, such as the potential pathways of reduced social support or diminished psychological resilience. These issues warrant further investigation.

## 5 Conclusion

The present study found that the negative association between caregiving competence and caregiving burden may be shaped by the structure of caregiving time. As the caregiving trajectory extended, the buffering effect of caregiving competence on burden showed a gradually declining trend, suggesting that the protective effect of competence may have a temporal boundary. Further analysis revealed that the key factor influencing this relationship was not the intensity of daily caregiving input, but rather the cumulative length of the caregiving trajectory. On this basis, the present study attempts to conceptualize the caregiving trajectory as a temporal mechanism variable for understanding caregiver burden, thereby expanding caregiving time from a singular quantitative indicator to an analytical dimension with structural meaning.

This finding offers a novel augmentation to models of the caregiving stress process. Existing research has largely treated caregiving time as a background variable or a linear cumulative indicator, with limited attention to how its varying temporal structures impact stress mechanisms. Our results indicate that the caregiving trajectory itself can act as a persistent depleting force, progressively exhausting resources and accumulating role-related stress, thereby attenuating the protective efficacy of caregivers' internal resources. This implies that the formation of caregiver stress is not only related to caregiving tasks and input intensity but also intrinsically linked to the sustained temporal trajectory of the caregiving role. From a public health perspective, this process reflects the chronic psychological stress generated by long-term family caregiving and its potential impact on caregiver health, highlighting the family caregiver population as a significant health risk group requiring focused attention.

These findings provide a complementary perspective to the caregiver stress process model. Existing studies have largely treated caregiving time as a background variable or a linear cumulative indicator, with limited attention to the effects of different temporal structures on stress mechanisms. The results of this study suggest that the caregiving trajectory itself may act as a sustained depleting factor, gradually eroding the protective efficacy of caregivers' internal resources through progressive resource depletion and cumulative role strain. This implies that the formation of caregiver stress is related not only to caregiving tasks and the intensity of caregiving input but also to the temporal trajectory of assuming the caregiving role. From a public health perspective, this process reflects the chronic psychological stress generated by long-term family caregiving and its potential impact on caregiver health, highlighting that family caregivers constitute a population at increased risk for health problems requiring focused attention.

The findings of this study have several implications for future research and clinical practice. (1) At the research level, longitudinal designs should be employed in future studies to further validate the influence of the caregiving trajectory on the relationship between caregiving competence and burden, and to explore the dynamic process through which the protective effect of competence changes over the course of caregiving. (2) At the practical level, continuous caregiving time could be incorporated into caregiver assessments to identify long-term caregivers at risk of psychological exhaustion. Beyond routine competence training, comprehensive interventions such as time-limited respite care, psychological support, and social services may be explored to alleviate the prolonged stress burden of family caregivers.

## Data Availability

The original contributions presented in the study are included in the article/supplementary material, further inquiries can be directed to the corresponding author.
